# A mixed-methods study to determine the impact of COVID-19 on food security, food access and supply in regional Australia for consumers and food supply stakeholders

**DOI:** 10.1186/s12937-022-00770-4

**Published:** 2022-03-20

**Authors:** Stephanie Louise Godrich, Johnny Lo, Katherine Kent, Flavio Macau, Amanda Devine

**Affiliations:** 1 School of Medical and Health Sciences, Edith Cowan University, Bunbury, WA 6230 Australia; 2 School of Science, Edith Cowan University, Joondalup, WA 6027 Australia; 3 School of Health Sciences, Western Sydney University, Campbelltown, NSW 2560 Australia; 4 School of Business and Law, Edith Cowan University, Joondalup, WA 6027 Australia; 5 School of Medical and Health Sciences, Edith Cowan University, Joondalup, WA 6027 Australia

**Keywords:** Food security, Food access, Regional food supply, COVID-19

## Abstract

**Background:**

The COVID-19 pandemic has impacted the Australian food supply through changed consumer purchasing patterns, and potentially, household food security. The aim of this study was to determine the impact of COVID-19 on the prevalence of food insecurity and food supply issues, and perspectives of food supply stakeholders in regional Australia.

**Methods:**

A mixed-methods consumer survey and in-depth interviews with food supply stakeholders were conducted in regional Australia, more specifically South West Western Australia between May and July 2020, immediately after the 1^st^ wave of the pandemic.

**Results:**

The prevalence of food insecurity was 21% among consumers, and significantly more prevalent for those aged less than 30 years and living with a disability. Most consumers (73%) agreed that the COVID-19 pandemic had impacted the food supply. Food insecure respondents were more likely to report that food was more expensive, resulting in changes to the types and quantities of food bought. Food supply stakeholders perceived that consumers increased their intention to buy locally grown produce. Panic buying temporarily reduced the availability of food for both food suppliers and consumers, regardless of their food security status.

**Conclusions:**

This study provided novel insights from South West Australian consumer and food supply stakeholder perceptions. Food insecure consumers provided insights about the high cost of food and the subsequent adaptation of their shopping habits, namely type and amount of food purchased. Stakeholder perceptions largely focused on supply chain issues and corroborated consumer reports.

**Supplementary Information:**

The online version contains supplementary material available at 10.1186/s12937-022-00770-4.

## Background

Australia is reportedly among the most food secure countries in the world, due to high levels of food production and food availability [1]. However, inequitable food access is indeed an issue in Australia, resulting in poor food security for some households. Food security is defined as “when all people, at all times, have physical and economic access to sufficient, safe and nutritious food to meet their dietary needs and food preferences for an active and healthy life” [2]. The absence of or disruption to the availability, access, utilisation of food and stability of the food supply indicates food insecurity [2]. The most recent national estimated prevalence of food insecurity in Australia, in 2011–2012, was approximately 4–5% [3]. However, some individuals with vulnerabilities are more likely to experience food insecurity and are defined as *“individuals who may be unable to protect themselves against harm or exploitation” *[4]. Australian studies in vulnerable people, such as younger adults, older adults, refugees, Aboriginal people, females and university students, have reported food insecurity prevalence between 2 to 90% depending on the tool used [5]. Several factors which impacted food insecurity included being homeless or living independently, higher levels of disadvantage, low income, poor educational attainment or having a higher number of dependent children.

The COVID-19 pandemic began impacting Australia in January 2020, and the first case was reported in Western Australia (WA) on the 1st March 2020. The pandemic temporarily disrupted parts of food supplies through changes in consumer behaviours and social distancing restrictions [6]. Food prices reportedly increased across the world resulting from a surge in demand with impacts felt for more than two years [7]. Food shopping was impacted by purchase limits on food items and reduced interactions with staff to maintain safety. The culmination of these effects is likely to impact food security. Unsurprisingly, food insecurity during COVID-19 in Tasmania, Australia was measured at 26% [8], substantially higher than pre-pandemic estimates in this region (6%) [9]. UK and USA food insecurity prevalence escalated from 14% to 24% (pre-pandemic) [10, 11] to up to 64% (during the pandemic), among low income groups [12].

Regional WA residents are at increased risk of food insecurity [13]. However, the prevalence during the COVID-19 pandemic is not known, and it is not well understood how the pandemic impacted food access and supply from the perspective of consumers and food supply stakeholders. In order to support future food security, it is important to understand how COVID-19 has affected food supply chains to inform potential solutions. Therefore, this study’s objectives were to (i) determine the prevalence of food insecurity in South West Western Australia (SWWA) during the COVID-19 pandemic; (ii) quantify the relationship between food supply issues and food security; and (iii) understand how the COVID-19 pandemic impacted food bought and consumed in regional Australia.

## Methods

The key food-producing region of SWWA spans approximately 24,000 square kilometres, located to the south of WA’s capital city Perth [14]. In 2019, there were 179,791 residents with the highest proportion reporting a household income $2,000-$2,499 per week [15]. An interpretive mixed-methods study was undertaken through a cross-sectional consumer survey and food supply stakeholder interviews. Ethics approval was received from the Edith Cowan University Human Research Ethics Committee (2019–00966-GODRICH).

### Consumer survey

A sample size calculation conducted via the Australian Bureau of Statistics website (https://www.abs.gov.au/websitedbs/d3310114.nsf/home/sample+size+calculator) determined a sample of *n* = 355 was required. Adult residents of SWWA were recruited through engagement of a professional list broker (postcodes 6172–6176 and 6207–6398), in addition to social media, local media and web-based advertisements. The inclusion criteria were South West region [16] residents aged 18 years and over. The list broker included quotas for age ranges in line with the Census Bunbury region [17] and a gender quota of 50% male and 50% female.

A 29-item survey was developed including closed-ended and open-ended response questions (Additional file 1). Briefly, the survey included the US Household Food Security Survey Module (HFSSM) Six-item Short Form using a 30-day reference period [18] and demographic measures informed by food security research conducted during COVID-19 [11]. Two questions related to the number of days’ worth of food stored in the household and whether the respondent tried to purchase food groups but were unavailable to them in the preceding 30 days. Respondents indicated their agreement on a 5-point Likert scale to fourteen statements based on the determinants of food security [19] about how COVID-19 had impacted food purchasing and consumption behaviours. Additionally, two open-ended questions asked respondents to describe their perceptions of how COVID-19 had impacted Australia’s food supply.

Data were collected from May 3^rd^ to July 14^th^ 2020 using Qualtrics (Qualtrics Software Version 2020, Copyright 2020, Provo, UT, USA). An information letter and consent form were provided and respondents provided consent to access the survey.

Survey data were exported into IBM SPSS Statistics for Windows, Version 26.0 (IBM Corp. Armonk, NY), cleaned and analysed. Missing values resulted in the removal of eight responses. Due to low cell counts, Likert scales categories were collapsed. Food security status was recoded to ‘food secure’ (high food security) or ‘food insecure’ (marginal, low or very low food security). The continuous ‘age’ variable was recoded into six categories. Cross tabulations with Chi-square tests explored associations between the demographic variables and statements relating to how COVID-19 had impacted purchasing and consumption behaviours (predictors), with food security status (outcome). Multivariable logistic regression modelling with forward stepwise selection assessed the association between the predictors and food security. Stepwise regression established the relationship between demographic variables and COVID-19 statements. The significance level in the final model was set at *p* < 0.05.

Qualitative responses from the consumer survey were analysed thematically using QSR NVivo 12 Pro along with interview data.

### Food supply stakeholder interviews

Interview participants were selected using a process similar to the survey recruitment. Stakeholders representing food production, government, freight/logistics, retail, hospitality and services (e.g. childcare, aged care) sectors and in roles including primary producers, farmers’ market managers, child care centre cooks, or freight drivers. Of the 145 participants invited, 29 consented to participate including seven primary producers, seven local government community development or environmental health staff, four retailers, three childcare coordinators or cooks, two aged care hospitality coordinators, two logistics or freight managers, two open-air/farmers’ market managers, and two community food workers. Due to device recording failure on two occasions, 27 interviews were analysed in sufficient detail, along with fieldwork notes.

An interview guide (Additional file 2) containing 10 questions and associated prompts was developed based on the Food and Agriculture Organization’s Sustainable Food Systems Concept and Framework [20]. Questions included the interviewee’s work role and time in role, how COVID-19 had impacted supply chains, perceived changes to consumer purchasing during the pandemic, important steps to maintain the food supply during the pandemic and innovations interviewees had observed in relation to the food supply.

Two project team members conducted interviews from May 11^th^ to August 13^th^ 2020 via telephone, recorded with interviewees’ permission. Interviews ranged from 20 to 80 min, with an average of 48 min. To ensure key points were captured, field notes were taken and a ‘research journal’ entry was completed after each interview.

Interviews were transcribed to Microsoft Word, de-identified, checked for accuracy and imported into QSR NVivo 12 Pro (QSR International Pty Ltd. Release 1.0, 2020). A thematic analysis strategy was employed. The data were initially coded by one team member where inductive nodes were created. Nodes were combined where they contained similar statements. Text-search queries assisted with identifying instances where a theme had been discussed by a respondent elsewhere and these statements were parallel coded [21]. A date-order ‘analysis journal’ of the development of nodes and themes was kept in memorandum form in NVivo. A coding framework was developed including a summary of nodes and containing exemplar quotes. Data analysis was undertaken concurrently with data collection until saturation, confirmed at 27 interviews. Another two interviews were conducted to ensure robustness. Codes were thoroughly checked by three team members. The coding frame and sub-themes were reviewed until unanimous agreement was reached. Recoding of the analysis process resulted in new interpretations shared by the research team. A ‘rich, thick’ description of the whole dataset was undertaken in order to ensure transferability.

## Results

### Consumer quantitative survey

A total of *n* = 115 adults completed the online survey. Consumer survey respondents were typically highly educated (87%), female (83%), married (72%), over 51 years old (61%), with no children living in their household (79%), with a disability (79%), and were the main household shopper (86%) (Table 1). Most respondents (79%) had high food security and 21% were classified as food insecure. Participants most frequently reported eating less than they felt they should (12.3%, *n* = 13), followed by cutting the size of their meals or skipping meals (10.2%, *n* = 11), and not eating despite being hungry (9.3%, *n* = 10).

**Table 1:** Respondent demographics from consumer survey

Consumer survey respondents		
**Variable**	Response categories	Number (Percentage)
**Age (Years)**	18–30	12 (11.3)
	31–50	29 (27.4)
	51 +	65 (61.3)
**Gender**	Male	18 (17.0)
	Female	88 (83.0)
**Adults in Household**	1	15 (14.0)
	2	70 (65.4)
	3 or more	22 (20.6)
**Children in Household**	No children	79 (79.0)
	One or more child	21 (21.0)
**Educational attainment**	Completed Primary or Secondary School	14 (13.2)
	Completed Technical or Further Education or University Degree	92 (86.8)
**Marital status**	Married	76 (72.4)
	Single or widowed/separated/divorced	29 (27.6)
**Employment status**	Full time work	39 (36.4)
	Part time work or student and working full or part time	25 (23.4)
	Retired, unemployed, employed but not working	43 (40.2)
**Disability or health condition**	Yes	85 (79.4)
	No	22 (20.6)
**Main Shopper in Household**	Yes	91 (85.8)
	No	15 (14.2)
**Food security status**	Food secure	87 (79.1)
	Marginal food secure	9 (8.2)
	Low food security	7 (6.4)
	Very low food security	7 (6.4)

The number of days of food stored in households showed similar distribution: 1–7 days (26.9%, *n* = 29), 8–10 days (28.7%, *n* = 31), 11–14 days (21.3%, *n* = 23), and 14 + days (23.1%, *n* = 25). No significant association was found between the number of days of food stored and food security (*p* = 0.898). The majority of the respondents (73.8%, *n* = 79) tried to purchase a food item that was unavailable in the preceding 30 days. Grain-based foods were most frequently unavailable (51.2%, *n* = 62), followed by meat (24.8%, *n* = 30), dairy (19.8%, *n* = 24), vegetables (11.6%, *n* = 14), fruits (8.3%, *n* = 10) and discretionary foods (2.5%, *n* = 3).

Most respondents agreed that COVID-19 impacted Australia’s food supply (72.9%, *n* = 86). A significantly higher proportion of food insecure participants agreed that COVID-19 impacted the type of food available, food price, quality, the amount and type of food purchased, where and how frequently they purchased food, the money available to purchase food, the way they had prepared and stored food, and their household food wastage (*p* < 0.05) (Table 2). In contrast, a greater proportion of food secure participants reported that COVID-19 had no impact on the food that they bought and consumed (37.3% vs 13%, *p* = 0.019).

**Table 2:** Response to food supply statements between food secure and food insecure consumers

Question	Response	Food Security Status n(%)		*p*-Value
		Food secure (*n* = 87)	Food insecure (*n* = 23)	
**Type of food available**	Strongly Agree or Agree	50 (60.2)	20 (87.0)	**0.011**
	Neutral Disagree or Strongly Disagree	33 (39.8)	3 (13.0)	
**Food prices**	Strongly Agree or Agree	48 (57.8)	22 (95.7)	** < 0.001**
	Neutral Disagree or Strongly Disagree	35 (42.2)	1 (4.3)	
**Quality of food available**	Strongly Agree or Agree	27 (32.5)	13 (56.5)	**0.002**
	Neutral Disagree or Strongly Disagree	55 (66.3)	10 (43.5)	
**Amount of food bought**	Strongly Agree or Agree	35 (42.2)	18 (78.3)	**0.002**
	Neutral Disagree or Strongly Disagree	48 (57.8)	5 (21.7)	
**Types of food bought**	Strongly Agree or Agree	39 (47.0)	20 (87.0)	** < 0.001**
	Neutral Disagree or Strongly Disagree	44 (53.0)	3 (13.0)	
**Where food was bought**	Strongly Agree or Agree	47 (56.6)	20 (87.0)	**0.005**
	Neutral Disagree or Strongly Disagree	36 (43.4)	3 (13.0)	
**Transportation**	Strongly Agree or Agree	13 (15.7)	6 (26.1)	0.265
	Neutral Disagree or Strongly Disagree	70 (84.3)	17 (73.9)	
**Frequency of food shopping**	Strongly Agree or Agree	47 (56.6)	22 (95.7)	** < 0.001**
	Neutral Disagree or Strongly Disagree	36 (43.4)	1 (4.3)	
**Money available to purchase food**	Strongly Agree or Agree	14 (16.9)	20 (90.9)	** < 0.001**
	Neutral Disagree or Strongly Disagree	69 (83.1)	2 (8.7)	
**Food preparation**	Strongly Agree or Agree	26 (31.3)	17 (73.9)	** < 0.001**
	Neutral Disagree or Strongly Disagree	57 (68.7)	6 (26.1)	
**Food storage**	Strongly Agree or Agree	22 (26.5)	13 (56.5)	**0.008**
	Neutral Disagree or Strongly Disagree	61 (73.5)	10 (43.5)	
**Food safety**	Strongly Agree or Agree	11 (13.3)	7 (30.4)	0.066
	Neutral Disagree or Strongly Disagree	72 (86.7)	16 (69.6)	
**Food waste**	Strongly Agree or Agree	12 (14.5)	12 (52.2)	** < 0.001**
	Neutral Disagree or Strongly Disagree	70 (84.3)	11 (47.8)	
**No effect due to Covid-19**	Strongly Agree or Agree	31 (37.3)	3 (13.0)	**0.019**
	Neutral Disagree or Strongly Disagree	52 (62.7)	20 (87.0)	

Accounting for sociodemographic factors (Table 3), respondents aged 18–30 years were six times more likely to be food insecure (*p* = 0.031), with 64% deemed food insecure as opposed to 7.5% for the 51 + age group. Respondents with a disability (38%) were also three times more likely to be food insecure (*p* = 0.024) as those without (17%). Reporting a change in the availability of money to buy food due to the pandemic resulted in a significant increase in food insecurity risk (*p* = 0.003). Those consumers who agreed COVID-19 impacted food price were significantly more likely to be food insecure (3% vs 31%, *p* = 0.048). Consumer respondents who agreed that COVID-19 impacted how frequently they shopped (31%) were significantly more likely to be food insecure (*p* = 0.040).

**Table 3:** Association between demographics and food supply factor statements by food security status

Predictor	Response	Univariable		Multivariable	
		Estimate (SE)	*p*-Value	Estimate (SE)	*p*-Value
**Adults in household**	1	1.00 (ref)		1.00 (ref)	
	2	-1.39 (0.77)	0.073	1.66 (1.89)	0.382
	3 or more	-1.98 (0.57)	0.001	0.15 (1.24)	0.901
**Age in years**	18–30	3.07 (0.87)	< 0.001	6.91 (3.2)	0.031
	31–40	1.41 (0.9)	0.115	2.37 (1.75)	0.175
	41–50	0.97 (0.87)	0.267	2.82 (1.66)	0.089
	51–60	1.47 (0.77)	0.055	2.97 (1.71)	0.082
	61 +	1.00 (ref)		1.00 (ref)	
**Disability**	Yes	1.09 (0.54)	0.041	3.71 (1.65)	0.024
	No	1.00 (ref)		1.00 (ref)	
**Food Price**	Agree	2.77 (1.05)	0.008	6.61 (3.35)	0.048
	Neutral/Disagree	1.00 (ref)		1.00 (ref)	
**Frequency of food shopping**	Agree	2.72 (1.05)	0.009	3.62 (1.76)	0.040
	Neutral/Disagree	1.00 (ref)		1.00 (ref)	
**Money available to purchase food**	Agree	3.96 (0.8)	< 0.001	3.46 (1.16)	0.003
	Neutral/Disagree	1.00 (ref)		1.00 (ref)	

### Consumer qualitative survey responses

Open-ended responses by consumers were used to confirm the food supply statement ratings and provided greater insight into the impact of COVID-19 on food supply, purchasing and consumption behaviours. Responses to the open-ended questions have been synthesised as follows.

- Food prices
  Consumers reported purchasing *“cheaper meals”* due to financial impacts and higher food prices. Retailers were criticised for increasing food prices to *“take advantage of shoppers”*. Food cost was dependent on the type of food and suppliers clarified that some price hikes were unrelated to the pandemic. Additionally, spending more of the household budget on food to buy larger amounts than usual was mentioned.
- Amount of food bought
  Consumers purchased more food than usual and preserved it in household refrigerators and freezers. Some reported that panic buying resulted in staple items, such as pasta, peas, flour and tinned potatoes being unavailable. Some shoppers reported buying small quantities of some foods that were in *“short supply”* while others reported buying more: *“Occasionally I would buy two instead of one, as you never knew when it was going to be available again”*. Consumers reported buying fresh, seasonal produce, decreasing pre-prepared food, wasting less food, and using longer lasting foods.
- Where consumers shopped
  Unique arrangements were reported including fast food outlets selling eggs, and schools selling ‘family meals’. Restaurants pivoted to takeaway options, giving more options to consumers. Local shops and producers thrived as consumers opted to purchase from roadside stalls, specialty shops and smaller outlets as opposed to *“the big supermarkets.”* This was motivated by altruistic (e.g. supporting local farmers) and safety reasons (e.g. avoidance of crowds). Consumers reported having to access numerous outlets due to a lack of availability and/or the purchase limits on staple food items.
- Frequency of shopping
  Consumer participants often moved from shopping *“every other day”* to once per week. Other consumers reported stockpiling food to reduce the frequency of food shopping. *“I used to go to the grocery store twice a week, and mostly buy small quantities of food. However, with two at-risk people at home, we had to limit our outings.”*

### Food supply stakeholder interviews

The impact of COVID-19 on consumer purchasing behaviours was further explored through food supply stakeholder interviews, which triangulated the consumer quantitative and open-ended responses. Most participants were from retail, production, and government sectors and had varied experience in their role (Table 4). The key themes determined by thematic analysis were: Practicality; Panic buying; Locally grown produce; Independent consumers; and Types of foods.

- Practicality
  Consumer reports detailing how COVID-19 impacted where they shopped for food were corroborated by food supply stakeholder interviews; consumers shopped closer to home, partly due to safety concerns. Stakeholders perceived consumers prioritising quick shopping, home-delivery or online ordering picking up at the store. Boxed produce or buying in bulk enabled consumers to shop less frequently. Easy-to-access, affordable services were a priority. Some small businesses increased their profits due to the uptake in takeaway meals during the pandemic.
  *“Because the restaurants, cafes had to close, there was a huge uptake in takeaway meals …some of the cafes reported that they actually found they had more, a lot more customers… some of them are actually saying it was it was better for their business.” (Local Government staff)*

**Table 4:** Respondent demographics from food supply stakeholder interviews

Food supply chain stakeholder respondents		
	Stakeholder sector and role	n(%)
**Sector**^**a**^	Production	7 (25.9)
	Government	5 (18.5)
	Freight/logistics	2 (7.4)
	Retail	8 (29.6)
	Hospitality	2 (7.4)
	Services (i.e. aged care, child care)	7 (25.9)
**Role**^**a**^	Primary producer	7 (25.9)
	Retailer, open-air/farmers’ market managers	8 (29.6)
	Hospitality business owner	2 (7.4)
	Local government community development or environmental health staff	5 (18.5)
	Institution coordinator or cook (childcare, aged care, social services)	7 (25.9)
	Logistics or freight manager	2 (7.4)
**Duration in role**	2 years or less	4 (14.8)
	3–5 years	6 (22.2)
	6–10 years	7 (25.9)
	11–15 years	4 (14.8)
	16–30 years	4 (14.8)
	31 + years	2 (7.5)

^a^Some respondents reported having multiple roles which aligned with multiple sectors

Stakeholder commentary reflected consumer reports of increased food prices, e.g.: *“from my end it cost me more. So, I have to put the price up.” (Primary Producer)* and *“Earlier we talked about …the price shifting and why that all happened… There's just not enough really coming out of the ground because of the … high demand.”* (Primary Producer). Some food supply stakeholders attributed the high prices to events preceding the pandemic, such as fires, drought and poor growing seasons, e.g.: *“There’s been recent droughts, even at the moment we're not getting much spinach… because they’ve have had storms locally” (Retailer)* and *“Cauliflowers were short but that had nothing to do with COVID. That was because of there was a crop failure” (Retailer).* Increased food prices did not subside, with food inflation still on the rise.

- Panic buying
  Consumer behaviour witnessed by food supply stakeholders included stocking up on food and bulk buying. This corroborated consumer reports in the open-ended questions. The types of products included shelf-stable items which were easy to store, as opposed to fresh produce.
  *“The panic buying led to a cycle where you would get a huge number of sales, where basically nobody could keep up in the supply chain for two or three days of product. And then everybody had their fridges full and then, so would buy nothing for 10 days. And then you'd go back into this panic buying routine again. And that happened for about four weeks.” (Primary producer)*
- Locally grown produce
  Food supply stakeholders reported consumers resisted buying imported, non-Australian food and prioritised local produce. Shortages in highly centralised supply chains resulted in empty shelves for mainstream retailers, benefiting decentralised local suppliers. Again, this reinforced consumer statements regarding buying from farm gates, local shops and producers. Some consumers consciously supported small farmers, though comments suggest this may have been for a short period of time.
  *“It's changed the way people even deal with us in respect to them being far more appreciative of what the farmers do and what we were doing during this period. So, there's definitely been a shift in attitude for people”. (Retailer)*
- Food independent consumers
  Food supply stakeholders perceived consumers were searching for alternative food networks, and were minimising food purchased in major supermarkets. Other strategies, also reported by consumer respondents, included storing more food than usual as a safety net and cooking food from scratch.
  *“A lot of people who started making their own bread and they were making cakes and they were doing a lot more cooking from scratch”. (Institution coordinator)*
- Types of food purchased
  Retailer interviewees commented that consumers purchased higher quality produce and hearty, healthier foods as strategies to increase their health. They were also more willing to purchase less familiar produce, increasing their tolerance of imperfect produce.
  *“It was just a general sort of buying more hearty foods, there was a little bit as well, people being a bit more imaginative, I think. If there wasn't something on the shelf that they needed, they’d just get something else.” (Retailer)*

## Discussion

This mixed-method study’s first objective was to determine the prevalence of food insecurity in SWWA during the COVID-19 pandemic. The prevalence of food insecurity was 21%, which is substantially higher than pre-COVID estimates, with a 2019 WA survey (using a single item questionnaire) reporting a food insecurity prevalence of 2.5% [22]. Our statistics are similar to other Australian regions during the pandemic (26%) [8]. We found that disability and younger age was significantly associated with food insecurity, which may relate to the disproportionate impact of the pandemic on employment for younger people [23]. In WA over 67% of young people were concerned about their financial situation, and 44% had reported a loss of income due to COVID-19, which likely impacted financial access to food for this group. Furthermore, increasing social isolation and difficulties getting to the shops could have contributed to food insecurity in those with a disability due to social distancing restrictions and the risk of infection [11].

Our second objective was to quantify the relationship between food supply issues and food security. Most respondents agreed that the COVID-19 pandemic had impacted food supply, disproportionately affecting respondents in low food security contexts. As such, food insecurity was associated with agreement that COVID-19 impacted the type of food available, food price, quality, the amount and type of food purchased, the location and frequency of food purchased, the money available to purchase food, storage, preparation and household food wastage. Food costs appear to have impacted food security, with respondents who agreed that food prices had changed were six times more likely to be food insecure. Also, 92% of food insecure respondents (versus 17% of food secure respondents) reported having less money available for food. The frequency of food shopping was a predictor of food security, which may relate to the limits placed on staple food items at supermarkets and that food insecure families may have needed to purchase foods in greater quantities than allowed. In addition, our study showed that food insecure respondents cut down on the amount of food they consumed as a coping strategy, which may partly reflect food unavailability during the peak of the crisis.

The third study objective was to understand how the COVID-19 pandemic impacted food bought and consumed in regional Australia. Consumer respondents reported that food suppliers inflated prices due to increased demand of foods. On a national level, food retailers were accused of passing increased food costs on to consumers [24]. Increased prices may have been partly attributable to inflation at a national level, which experienced the highest growth since 2011 [25]. On the other hand, food supply stakeholder interviewees often mentioned prices did not change due to the pandemic but were conscious of environmental issues (e.g. bushfires) influencing food prices. However, some of those issues (e.g. higher transportation costs) can be related to the broader effect of the pandemic in global supply chains.

In regional Australia, food costs rise, quality drops, and availability declines with increasing distance from major cities [26], which could have further exacerbated food affordability in our study. The culmination of food prices [7] and lower availability of foods may have resulted in consumers purchasing less healthy, shelf-stable alternatives. Interestingly, our study found a relatively high availability of discretionary foods in comparison to fresh food groups, highlighting that accessing healthy foods may have become more challenging. The sudden and extreme changes in consumer shopping behaviours during the pandemic created imbalances in supply and demand and reduced the availability of food [27]. As such, consumers not only changed what they eat, but where and how they bought their food during COVID-19 [28, 29]. In our study, consumers reported shopping at multiple locations, often as a result of limited stock. Food supply stakeholders perceived consumers were shopping less frequently, opting to shop quickly, and preferring to use online and delivery options. While international studies have reported an increase in online shopping [30], in Australia both delivery and pick up services were initially suspended by major supermarkets, but not smaller outlets. Consequently, consumers reported purchasing from smaller supermarkets, roadside stalls and regional wholesalers. In response to social distancing restrictions limiting their usual trading options, many local businesses pivoted toward online platforms and delivery box schemes [31].

In our study, 47% of food secure and 87% of food insecure respondents reported buying different types of food, with increased consumer resourcefulness reported through home cooking. The increased flexibility and creativity in consumers’ shopping and cooking habits may relate to a greater amount of time spent at home and the closure of many hospitality businesses. Food supply stakeholders also suggested that fresh produce was a priority, and Australian supermarkets relaxed specifications for fresh produce in response to increased consumer demand [32], which benefited local producers. The increased consumption of fresh produce is reflected in international literature with nearly a quarter of survey respondents in Italy reporting an increased consumption of fruit and vegetables during a lockdown [33].

While the majority of respondents (73%) tried to purchase a food item that was unavailable, it is unclear to what extent this contributed to food insecurity. A UK study reported that food unavailability contributed to 40% of food insecurity experienced [11]. Personal stress and the perceived fear of food shortages contributed to food hoarding, which is a common reaction to manage the uncertainty of the food supply [34]. In general, our consumer respondents reported purchasing more food than usual during the pandemic, corroborating other studies [28, 35]. Consumers and food supply stakeholders both observed that some purchases were related to panic buying rather than a need for food. Following periods of panic buying consumers have reportedly sought information on food storage methods, including freezing and canning [36]. However, food supply stakeholders in our study perceived that excess food may be wasted.

Consumers sought locally grown food options as they trusted and wanted to support local producers. Previous research in SWWA shows the vast majority of consumers think locally grown food is very important [13]. Our analysis identifies an opportunity for local producers be responsive towards pivoting to serve their communities when food supplies are challenged [28]. However, it would be insufficient to rely on the adaptive capacity of small-scale producers, and therefore greater innovation may be required to ensure the resilience of food supply chains in regional Australia in the future.

This study has a number of strengths. To the authors knowledge, it is the first study to combine consumer and food supply stakeholder perspectives on how the COVID-19 pandemic has affected food supply and food security, providing triangulated data. This compliments recent Australian research which examined food chain resilience during the pandemic from an industry perspective [37]. The limitations include a small sample size, despite using a number of recruitment strategies, contributing to a potentially underpowered study, along with limited generalisability beyond the SWWA region. Additionally, a larger sample of stakeholders from transport and logistics sectors may have provided further insights as these were central to food supply chains during the pandemic.

## Conclusion

In conclusion, our study demonstrates that significant changes in consumer behaviours led to food supply issues and exacerbated food insecurity in vulnerable groups. Additionally, our study highlights several critical points in the Australian regional food supply during the COVID-19 pandemic which should inform improvements to food supply practices to maintain food security. We recommend that further support and improvements in social safety nets and disability support strategies are required to minimise the impacts of food insecurity for young people and those living with a disability. Communication through a variety of means will ensure vulnerable people are adequately informed about where they can access food, specific shopping hours, locations, and alternative food sourcing/delivery systems. Governments and media must improve communication about COVID-safe shopping options, the adequacy of the regional Australian food supply and equitable responses to food shopping to mitigate repercussions of panic buying. Lastly, stronger contingency arrangements to maintain the food supply in regional areas should be implemented learning from industries which have remained resilient throughout the pandemic [37], such as red meat and processed dairy. Our study indicates that systemic changes which sustain local food production, distribution, and promotion should be a priority. Recommendations on the supply side include: supermarkets to quickly implement buying limits, with agile decisions made at the local store rather than waiting for a directive from the central office; local food producers to be more open to innovative online/delivery models, supported by local governments; expand regional networks to monitor, escalate, and mitigate issues of concern. Government strategies to support access to sufficient, safe and healthy food for these groups for these groups should be a priority throughout the pandemic, including supporting financial access through increasing government safety nets. Additionally, government COVID-19 responses should be disability-inclusive [38] and prioritise strengthening social support mechanisms through disability service providers.

## Supplementary Information

**Additional file 1.**

**Additional file 2.**

## Data Availability

The datasets generated and analysed during the current study are not publicly available due to ongoing data analysis but are available from the corresponding author on reasonable request.
