# Supplementary material for: A mixed-methods study to determine the impact of COVID-19 on food security, food access and supply in regional Australia for consumers and food supply stakeholders
Source: Nutr J. 2022 Mar 20;21:17. doi: 10.1186/s12937-022-00770-4 (PMC8934375; doi:10.1186/s12937-022-00770-4)
Supplement: Supplementary file 1 — Additional file 1. [file 12937_2022_770_MOESM1_ESM.docx]

## In your opinion, has COVID-19 impacted Australia’s food supply?

- Yes (skip logic to q2)
- No (skip logic to q3)

## In what ways do you think COVID-19 has impacted Australia’s food supply?

*Open ended text box*

## Have you observed any innovative ways of maintaining the Australian food supply during COVID-19? (for example, businesses changing the way they sell food) If so, what are they?

*Open ended text box*

## Please indicate your level of agreement with the following statements about how COVID-19 has impacted the food you have bought and consumed:

| ***COVID19 has impacted…*** | **Strongly Disagree**  5 | **Disagree**  4 | **Neither agree or disagree**  3 | **Agree**  2 | **Strongly Agree**  1 |
| --- | --- | --- | --- | --- | --- |
| the type of food available |  |  |  |  |  |
| - the price of food |  |  |  |  |  |
| - the quality of food available |  |  |  |  |  |
| - the amount of food I have bought |  |  |  |  |  |
| - the type of foods I have bought |  |  |  |  |  |
| - where I have bought food from (i.e. different food outlets, different suburb/town, farm-direct, online) |  |  |  |  |  |
| - how I can get to the shops (i.e. transport) |  |  |  |  |  |
| - how frequently I have bought food |  |  |  |  |  |
| - the money I have had available to buy food |  |  |  |  |  |
| - the way I have prepared/cooked food |  |  |  |  |  |
| - the way I have stored food |  |  |  |  |  |
| - the safety of some foods |  |  |  |  |  |
| - The amount of uneaten food my household has thrown out/wasted |  |  |  |  |  |
| - COVID-19 has not impacted the food I have bought and consumed |  |  |  |  |  |

##

## Can you describe how COVID-19 has impacted the food you have bought and consumed, in relation to the reason/s you indicated above? For example, how has it changed where you have bought food?

*Open ended text box*

## How could South West WA’s food system be better prepared for a disaster in the future?

*Open ended text box*

## Approximately, how many days would the food you currently have stored in your house last your household?

- 1-3 days
- 4-7 days
- 8-10 days
- 11-14 days
- 14+ days

1. Below are several statements that people have made about their food situation. For these statements, please tell us whether the statement was often true, sometimes true, or never true for (you/your household) in the last 30 days.

The first statement is:

1. “The food that (I/we) bought just didn’t last, and (I/we) didn’t have money to get more.” Was that often, sometimes, or never true for (you/your household) in the last 30 days?

- Often true
- Sometimes true
- Never true
- Don’t know

1. “(I/we) couldn’t afford to eat balanced meals.” Was that often, sometimes, or never true for (you/your household) in the last 30 days?

- Often true
- Sometimes true
- Never true
- Don’t know

1. In the last 30 days, did (you/you or other adults in your household) ever cut the size of your meals or skip meals because there wasn't enough money for food?

- Yes
- No (Skip question 8ci – skip logic to Q8d)
- Don’t know (Skip question 9d – skip logic to Q8d)
  1. In the last 30 days, how many days did this happen?
- _______________ days
- Don’t know

1. In the last 30 days, did you ever eat less than you felt you should because there wasn't enough money for food?

- Yes
- No
- Don’t know

1. In the last 30 days, were you every hungry but didn't eat because there wasn't enough money for food?

- Yes
- No
- Don’t know

## In the last 30 days, have you wanted to buy a food item that was unavailable in the shop?

- Yes
- No (skip logic to q11)

## What types of foods did you try to buy that were unavailable?

*(tick all that apply)*

- Fruit
- Vegetables
- Grain-based foods (bread, pasta, rice)
- Meat or other protein foods (chicken, eggs, tofu, lentils)
- Dairy foods (milk, yoghurt, cheese)
- Junk foods (i.e. pizza, confectionary)
- Not applicable

# Perceptions of South West Western Australian fruit and vegetable produce

## How important do you believe buying South West WA grown produce is?

- Very important
- Fairly Important
- Important
- Slightly Important
- Not at all important
- No opinion

1. Has your opinion about importance of South West WA grown produce changed as a result of COVID-19?

- Yes, I think it is more important
- Yes, I think it is less important
- No, my opinion is the same
- I don’t have an opinion

## Has your ability to buy South West Western Australian grown produce changed as a result of COVID-19? Have you bought:

- Significantly more
- Somewhat more
- The same
- Somewhat less
- Significantly less

## Where have you bought South West Western Australian grown produce as a result of COVID-19?

*(tick all that apply)*

- Unsure/I have not bought South West WA grown produce
- Major supermarkets (i.e. Coles, Woolworths)
- Independent or minor supermarkets (i.e. IGA)
- General or corner stores
- Specialty shops
- Fruit and vegetable shops
- Local farmers markets
- Farm gate sales
- Grown your own
- Home delivery/box schemes

## Approximately, what percentage of the food you have bought since COVID-19 has been South West WA grown?

- 0-25%
- 26-50%
- 51-75%
- 76-100%
- Unsure

## Age

- 18-30
- 31-40
- 41-50
- 51-60
- 61+

## Sex

- Male
- Female

## Education

- Primary
- Secondary
- Tertiary (undergraduate, TAFE)
- Tertiary (postgraduate)

## Martial Status

- Married or Defacto
- Single
- Separated/Widowed/Divorced

## Adults in household

- 1
- 2
- 3 or more

## Children in household

- 0
- 1
- 2
- 3 or more

1. Postcode ______________
2. Suburb/Town name _______________

## What is your employment status (within the past 3 months)? (multiple response option)

- Working full time
- Working part time
- Full time student
- Retired
- Unemployed
- Not working/other

## Has your employment status changed since 1^st^ March 2020?

- No
- Yes, gained employment
- Yes, reduced employment
- Yes, lost employment

## Household income

- <20,000-40-000
- 40,000 – 60,000
- 60,000-80,000
- 80,000 – 100,000+

1. Have you experienced a drop in income as a result of the COVID-19 crisis?

- No loss in income
- Up to a 24% drop in income
- 25% to 49% drop in income
- 50% to 74% drop in income
- 75% to 99% drop in incom
- We have lost all of our income
- Don't know/prefer not to say

## Are you the main shopper for your household?

- Yes
- No

1. Do you have a health condition or disability that limits your activity:

- No
- Yes, a lot
- Yes, a little
