# Supplementary material for: A mixed-methods study to determine the impact of COVID-19 on food security, food access and supply in regional Australia for consumers and food supply stakeholders
Source: Nutr J. 2022 Mar 20;21:17. doi: 10.1186/s12937-022-00770-4 (PMC8934375; doi:10.1186/s12937-022-00770-4)
Supplement: Supplementary file 2 — Additional file 2. [file 12937_2022_770_MOESM2_ESM.docx]

1. Could we start by you telling me a little bit about your work role?
2. How many months/years have you been in this role?
3. Could you provide me with a **picture of** what **South West WA food supply chains** (the ways of getting food from producers to consumers) were like before COVID-19?
   1. *Prompt: What key food supply chains were used / key ways of selling/purchasing food?*
   2. *Prompt: How environmentally-friendly were they? (i.e. the way they were transported, short or long distance)*
   3. *Prompt: Which food supply chains brought the highest economic return to producers, do you think?*

**Moving onto COVID-19…**

1. How has COVID-19 impacted South West WA food supply chains – either negatively and/or positively?
   1. *Prompt: Have the kinds of supply chains producers normally use changed? If so, how?*
   2. *Prompt: how has it changed jobs and profits in the food supply industry, do you think?*
   3. *Prompt: Has COVID-19 impacted food loss/waste, do you think? If so, how?*
2. What about consumers? Can you describe any perceived changes in consumer purchasing behaviour as a result of COVID-19? If so, how?
   1. *Prompt: Where do you see the biggest difference in attitude occurring?*
   2. *Prompt: What key perceptions and concerns (i.e. scarcity? Safety? Health?) are factors?*
   3. *Prompt: how has it changed where, how and what food people are buying?*
   4. *Prompt: What are some impacts of COVID-19 on equity, for example, for all people to access food?*

**Barriers and enablers…**

1. What are some **barriers** to relying more on short food supply chains (i.e. farmers’ markets, farmgate sales, Community Supported Agriculture) that are a result of the pandemic?
2. What are some **enablers** **or opportunities** associated with relying more on short food supply chains that are a result of/have come from the pandemic? (i.e. borders closing, intrastate travel restricted)
3. What, in your opinion, have been the most important steps taken during the pandemic, to maintain the food supply?

**Innovations and future vision…**

1. What innovations have you heard of or seen, in relation to the food supply, since the start of COVID-19?
   1. *Prompt: What key learnings can we take from the pandemic?*
   2. *Prompt: how can we increase the resiliency of the food supply chains in the future through innovations?*
2. Thinking forward to a year from now, after the pandemic, what would you like our South West food supply chains to look like?
   1. *Prompt: what supply chains will be used, what will consumer purchasing/preferences look like?*
   2. *Prompt: What recommendations do you have for us to achieve this vision?*

Is there anyone else I should interview?

Thank you very much for your time and insights today!
